# Supplementary material for: The effect of intramuscular injection technique on injection associated pain; a systematic review and meta-analysis
Source: PLoS One. 2021 May 3;16(5):e0250883. doi: 10.1371/journal.pone.0250883 (PMC8092782; doi:10.1371/journal.pone.0250883)
Supplement: S1 Table — *The coefficient estimates how the intervention effect (SMD) differs (RCT studies vs Quasi-experimental studies). (DOCX) [file pone.0250883.s002.docx]

**S1 Table. Meta-regression of RCT (n=3) and quasi-experimental studies (n=7) on the intervention effect of IMI techniques applying pressure to the IMI site**

| **Parameter** | **Coefficient* (95%CI)** | **t value** | **P value** |
| --- | --- | --- | --- |
| RCT studies | -0.24 (-2.71, 2.23) | -0.22 | 0.829 |

*The coefficient estimates how the intervention effect (SMD) differs (RCT studies vs Quasi-experimental studies)
