# Supplementary material for: The effect of intramuscular injection technique on injection associated pain; a systematic review and meta-analysis
Source: PLoS One. 2021 May 3;16(5):e0250883. doi: 10.1371/journal.pone.0250883 (PMC8092782; doi:10.1371/journal.pone.0250883)
Supplement: S2 Table — Ŧ- excludes Barnhill et al. [34], Zore and Dias [39], Khanra et al. [22] and Rani and Prasad [21]. (DOCX) [file pone.0250883.s003.docx]

**S2 Table. Sensitivity analyses: Local pressure IMI techniques**

| **Meta-analysis** | **Number of studies** | **Pooled SMD(95%CI)** | **P value** | **Heterogeneity (95%CI)** |
| --- | --- | --- | --- | --- |
| Pressure on IMI site Studies | 10 | -1.44 (-1.99,-0.89) | >0.001 | I^2^ =95% (92,97) |
| Pressure on IMI site Studies (ignoring cross-over designs) | 10 | -1.51 (-2.19, -0.83) | >0.001 | I^2^ =97% (95,98) |
| Pressure on IMI site Studies (Fixed effects) | 10 | -0.74 (-0.86, -0.63) | >0.001 | I^2^ =95% (92,97) |
| Pressure on IMI site Studies (excluding small studies)Ŧ | 6 | -1.00 (-1.56, -0.44) | >0.001 | I^2^ =94% (90;97) |

Ŧ- excludes Barnhill et al [34], Zore and Dias[39], Khanra et al [22] and Kanika and Rani [21]
