# Supplementary material for: The effect of intramuscular injection technique on injection associated pain; a systematic review and meta-analysis
Source: PLoS One. 2021 May 3;16(5):e0250883. doi: 10.1371/journal.pone.0250883 (PMC8092782; doi:10.1371/journal.pone.0250883)
Supplement: S3 Table — Ŧ–includes Najafidolatbad et al. [27] study (Z track vs air lock technique). (DOCX) [file pone.0250883.s004.docx]

**S3 Table. Sensitivity analyses: Z track IMI technique**

| **Meta-analysis** | **Number of studies** | **Pooled SMD(95%CI)** | **P value** | **Heterogeneity (95% CI)** |
| --- | --- | --- | --- | --- |
| Included Z track based Studies | 2 | -0.20 (-0.41,0.01) | 0.060 | I^2^ =0% (no estimate) |
| Included Z track based Studies (ignoring cross-over design) | 2 | -0.22 (-0.49, 0.07) | 0.120 | I^2^ =0% (no estimate) |
| Included Z track based Studies (Fixed effects) | 2 | -0.20 (-0.41,0.01) | 0.060 | I^2^ =0% (no estimate) |
| All Z track intervention studies ^Ŧ^ | 3 | 0.22 (-0.64, 1.09) | 0.612 | I^2^ =93% (84,97) |

Ŧ – includes Najafidolatbad et al[27] study (Z track vs air lock technique) .
