# Supplementary material for: The effect of intramuscular injection technique on injection associated pain; a systematic review and meta-analysis
Source: PLoS One. 2021 May 3;16(5):e0250883. doi: 10.1371/journal.pone.0250883 (PMC8092782; doi:10.1371/journal.pone.0250883)
Supplement: S4 Table — (DOCX) [file pone.0250883.s005.docx]

**S4 Table. Sensitivity analyses: Acupressure IMI technique**

| **Meta-analysis** | **Number of studies** | **Pooled SMD(95%CI)** | **P value** | **Heterogeneity (95%CI)** |
| --- | --- | --- | --- | --- |
| Acupressure Studies | 4 | -1.62 (-2.80,-0.44) | 0.007 | I^2^ =96% (92,98) |
| Acupressure Studies (ignoring cross-over design) | 4 | -1.63 (-2.74, -0.52) | 0.004 | I^2^ =96% (93,98) |
| Acupressure Studies (Fixed effects) | 4 | -1.46 (-1.70,-1.22) | >0.001 | I^2^ =96% (92,98) |
| Acupressure studies (Ignoring the L14 pressure point intervention) | 4 | -1.65 (-2.79, -0.52) | 0.004 | I^2^ =95% (90,97) |
