# Supplementary material for: The effect of intramuscular injection technique on injection associated pain; a systematic review and meta-analysis
Source: PLoS One. 2021 May 3;16(5):e0250883. doi: 10.1371/journal.pone.0250883 (PMC8092782; doi:10.1371/journal.pone.0250883)
Supplement: S5 Table — (DOCX) [file pone.0250883.s006.docx]

**S5 Table. Sensitivity analyses: Cold needle IMI technique**

| **Meta-analysis** | **Number of studies** | **Pooled SMD(95%CI)** | **P value** | **Heterogeneity (95%CI)** |
| --- | --- | --- | --- | --- |
| Cold needle studies | 2 | -0.73 (-1.83, 0.37) | 0.194 | I^2^ =92% (72,98) |
| Cold needle studies (ignoring cross-over design) | 2 | -0.74 (-1.84, 0.36) | 0.187 | I^2^ =94% (81,98) |
| Cold needle studies (Fixed effects) | 2 | -0.73 (-1.04,-0.42) | >0.001 | I^2^ =92% (72,98) |
