# Supplementary material for: The effect of intramuscular injection technique on injection associated pain; a systematic review and meta-analysis
Source: PLoS One. 2021 May 3;16(5):e0250883. doi: 10.1371/journal.pone.0250883 (PMC8092782; doi:10.1371/journal.pone.0250883)
Supplement: S6 Table — (DOCX) [file pone.0250883.s007.docx]

**S6 Table. Sensitivity analyses: Choice of gluteal IMI site**

| **Meta-analysis** | **Number of studies** | **Pooled SMD(95%CI)** | **P value** | **Heterogeneity (95%CI)** |
| --- | --- | --- | --- | --- |
| Studies on ventrogluteal vs dorsogluteal IMI site | 2 | -0.43 (-0.81, -0.06) | 0.024 | I^2^ =0% (no estimate) |
| Studies on ventrogluteal vs dorsogluteal IMI site (ignoring cross-over design) | 2 | -0.43 (-0.71, -0.15) | 0.003 | I^2^ =0% (no estimate) |
| Studies on ventrogluteal vs dorsogluteal IMI site (Fixed effects) | 2 | -0.43 (-0.81, -0.06) | 0.024 | I^2^ =0% (no estimate) |
