# Supplementary material for: The effect of intramuscular injection technique on injection associated pain; a systematic review and meta-analysis
Source: PLoS One. 2021 May 3;16(5):e0250883. doi: 10.1371/journal.pone.0250883 (PMC8092782; doi:10.1371/journal.pone.0250883)
Supplement: S7 Table — *Asterisk used to capture multiple word endings (e.g. technique, techniques). (DOCX) [file pone.0250883.s008.docx]

**S7 Table. Search terms in abstract and title**

| Steps | Search terms |
| --- | --- |
| 1 | Intramuscular injection OR intra-muscular injection |
| 2 | Technique* |
| 3 | 1 AND 2 |

* Asterisk used to capture multiple word endings (e.g. technique, techniques)

**Limits**

| Date range | Language | Material type |
| --- | --- | --- |
| All years (inception to June 29, 2020) | Any language | Human studies |
